# Active Food Packaging Made of Biopolymer-Based Composites

**DOI:** 10.3390/ma16010279

**Published:** 2022-12-28

**Authors:** Xuanjun Hu, Chao Lu, Howyn Tang, Hossein Pouri, Etienne Joulin, Jin Zhang

**Affiliations:** 1 Department of Chemical and Biochemical Engineering, University of Western Ontario, London, ON N6A 5B9, Canada; 2 School of Biomedical Engineering, University of Western Ontario, London, ON N6A 5B9, Canada

**Keywords:** active packaging, antimicrobial packaging, biopolymers, metal oxide nanoparticles, composites, polyphenols, antibacterial mechanism

## Abstract

Food packaging plays a vital role in protecting food products from environmental damage and preventing contamination from microorganisms. Conventional food packaging made of plastics produced from unrenewable fossil resources is hard to degrade and poses a negative impact on environmental sustainability. Natural biopolymers are attracting interest for reducing environmental problems to achieve a sustainable society, because of their abundance, biocompatibility, biodegradability, chemical stability, and non-toxicity. Active packaging systems composed of these biopolymers and biopolymer-based composites go beyond simply acting as a barrier to maintain food quality. This review provides a comprehensive overview of natural biopolymer materials used as matrices for food packaging. The antioxidant, water barrier, and oxygen barrier properties of these composites are compared and discussed. Furthermore, biopolymer-based composites integrated with antimicrobial agents—such as inorganic nanostructures and natural products—are reviewed, and the related mechanisms are discussed in terms of antimicrobial function. In summary, composites used for active food packaging systems can inhibit microbial growth and maintain food quality.

## 1. Introduction

Foodborne illnesses, commonly known as food poisoning, are caused by food contamination with bacteria, viruses, parasites, and other chemical hazards, such as heavy metals [1,2,3]. Foodborne illnesses mainly cause gastrointestinal issues, ranging from diarrhea and vomiting to cancers, and they can also produce severe neurological, gynecological, and immunological symptoms [4]. Such illnesses not only contribute to great food safety and insecurity issues across the world, but also pose a great threat to public health and lead to substantial economic losses. According to a WHO investigation, approximately one-tenth of people worldwide contract foodborne illnesses after consuming contaminated food, which could lead to over 420,000 deaths each year [4]. In addition, some types of foodborne illnesses caused by microorganisms, such as listeriosis, are particularly harmful to immunocompromised populations such as children and pregnant women [5]. In all stages of the food supply chain, including production, transportation, and consumption, food can be contaminated due to environmental contamination and cross-contamination during food handling. Therefore, to reduce the risks of foodborne illnesses, it is essential to prevent food contamination in the food supply chain. Food packaging acts as a physical barrier to prevent food contamination. The need for active packaging is urgent due to the increasing demand for safe and high-quality food products.

Traditional food packaging materials are designed to avoid unwanted interactions with food [6]. With the development of nanotechnology, the packaging industry has evolved from traditional packaging to active packaging. Active packaging can go beyond simply acting as a barrier. Due to its antimicrobial effects against pathogens and vegetative spoilage bacteria, it has been effective in preventing foodborne illnesses, extending shelf life, and maintaining food quality [7]. In addition, oxygen, ethylene scavengers, moisture absorbers, carbon dioxide releasers, and antimicrobial packaging systems are typical examples that can be categorized into active packaging systems [7]. Scavenging systems can absorb harmful compounds from the food surface or from the headspace within the packaging. 

Plastic materials—such as poly(vinyl chloride) (PVC), polyethylene terephthalate (PET), polypropylene (PE), and polypropylene (PP)—have been widely applied in food packaging for many decades because of their superior barrier properties against water and oxygen and their relatively low cost [8,9]. However, these petrochemical-based plastic materials are produced from non-renewable fossil fuels and are difficult to recycle and degrade at a fast rate, posing a great threat to the environment. Biopolymer-based materials have demonstrated their great potential due to their abundance in nature, degradability, and antimicrobial properties; chitosan would be a typical example. Nanomaterials and nanocomposites in the packaging can maintain food quality and freshness by preventing microbial activity with antimicrobial agents, as well as preventing lipid oxidation and rancidity by maintaining a low-oxygen atmosphere within the packaging. A variety of antimicrobial agents can be integrated into packaging materials to be slowly released and inactivate the microorganisms efficiently. These nanocomposites can even be incorporated into oxygen scavengers to control the packaging’s inner environment, thereby inhibiting the growth of aerobic bacteria and molds instead of utilizing antimicrobial systems [10]. 

**Figure 1:**
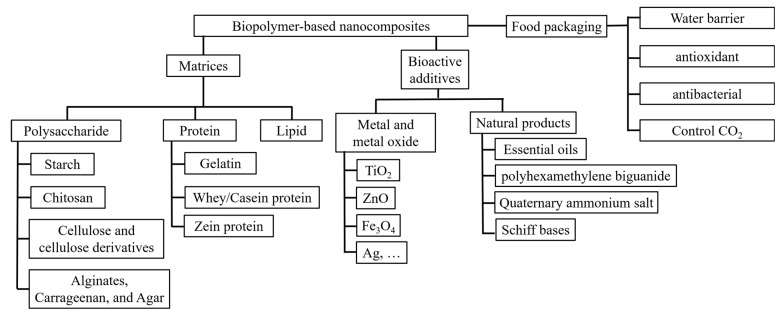
Components of biopolymer-based nanocomposites and their properties for food packaging.

In this review, the components of biopolymer-based nanocomposites and their applications for food packaging are summarized and discussed to show their great potential for active food packaging. The discussion of the components is separated into two parts: polymeric matrices, and additives with antimicrobial properties. Various polymers—such as polysaccharides (including starch, chitosan, and cellulose), proteins (including gelatin, whey, and zein), and lipids—are summarized to show their possibilities for the film matrices and related reinforcement strategies. Their antioxidant, water barrier, and oxygen barrier properties are also discussed in the following sections. After that, the metal and metal oxide nanoparticles, bioactive natural additives (e.g., essential oils, Schiff bases, polyphenols), and related antimicrobial mechanisms are discussed in the next section. Then, the discussion focuses on how to use gas releasers and absorbers in packaging systems to control carbon dioxide (as shown in Figure 1). Finally, insight into the emerging practical active food packaging systems is provided.

## 2. Natural Biopolymers Used as the Matrices of Food Packaging

Currently, most plastic packaging used in the industry is produced from petrochemical-based materials because of their ease of processing, low cost, wide availability, good mechanical properties, and barrier properties [11]. However, these conventional plastic materials are difficult to recycle and degrade, and their disposal contributes to severe environmental pollution [12]. Therefore, bio-based materials—including polysaccharide-, protein-, and lipid-based materials [13]—would be great alternatives to conventional petrochemical-based plastics, especially in the food industry, where a great amount of single-use and short-lived packaging is used. In addition, biopolymer materials could be degraded through the metabolism of fungi or other microorganisms, under appropriate conditions (e.g., oxygen, moisture, and temperature). They can be decomposed into inorganic biomass such as CO_2_, H_2_O, and CH_4_ with no harmful residues [12]. Biopolymer-based materials may be safer than conventional plastic packaging materials with respect to food safety concerns because of the biocompatibility of the materials and the reduced use of industrial plasticizers. However, the existing research on the application of biopolymers in food packaging is still in the early stages because of their relatively poor mechanical properties, low resistance to heat, and bad barrier properties [12]. Several methods have been developed to improve these properties for food packaging, such as the addition of plasticizers and nanomaterials, chemical modification of polymers, and gamma irradiation treatment [14,15]. These materials can be categorized as polysaccharide-based (starch, chitosan, cellulose, etc.), protein-based (such as gelatin, whey, and zein), and lipid-based biopolymers.

### 2.1. Polysaccharide-Based Biopolymers

Polysaccharides are one of the most common and abundant types of biopolymers from natural sources (such as marine crustaceans [16] or plant fibers [17]). They are increasingly being applied as novel packaging materials due to their wide abundance, easy availability, and non-toxicity. Polysaccharide-based packaging materials also exhibit excellent CO_2_ and O_2_ barrier properties, which can not only retard the respiration rate of food but also potentially inhibit the growth of pathogens, spoilage bacteria, and molds within the packaging [6]. 

#### 2.1.1. Starch

Starch is a naturally renewable polysaccharide-based biopolymer derived from crops (e.g., maize, corn, rice, potatoes, soy, etc.) [13]. It has various attractive attributes as a novel food packaging material, such as non-toxicity and cost-effectiveness due to its wide availability and ready accessibility. Amylose (a linear molecule with few branches) and amylopectin (a branched-chain molecule) are two constituents of starch, in which amylose plays an important role in film-forming [6,18]. The presence of amylose tends to cause the formation of a coherent and strong film, while starch with a higher amylopectin content forms a brittle film with lower tensile strength [6,19]. Therefore, starch with higher amylose contents exhibits better thermoplastic behavior and should be preferred for the production of thermoplastic starch as a packaging material [18]. Starch-based films also show good oxygen barrier properties, potentially preventing microbial growth. However, the hydrophilic nature of starch-based films causes them to have poor moisture barrier properties and inferior mechanical properties [6,13]. Surface modification and the incorporation of plasticizers, nanoparticles, or other biopolymers can be used to enhance the mechanical properties of starch-based packaging materials [11]. For example, incorporating starch with chitosan can decrease the water sensitivity and enhance the tensile strength of the film-forming material [13]. Antimicrobial agents can also be added to achieve a desired antimicrobial effect.

#### 2.1.2. Chitosan

Chitosan is the deacetylated chitin with the molecular structure of a linear amino polysaccharide and is abundant in animals such as crustaceans (e.g., shrimp, crabs, and lobster) [20,21]. Typically, chitosan cannot be dissolved in neutral or alkaline water, but it can be dissolved in diluted acetic acid and hydrochloric acid solutions [21]. The solubility of chitosan in weak acids is the key to its film-forming ability. Coating, nanoparticles, and casted films are the main methods to improve chitosan-based films utilized in packaging [13]. Chitosan is a biopolymer that naturally possesses antimicrobial properties against various microorganisms (including *Actinomyces*, *Clostridium*, *Mycobacterium*, *streptococci*, *staphylococci*, *Nocardia Pseudomonas*, *Salmonella*, and *Escherichia* [22]). Both molecular weight and degree of acetylation affect the antimicrobial abilities of chitosan [23]. Therefore, chitosan films can be widely applied to preserve a variety of foods and maintain their quality.

#### 2.1.3. Cellulose and Cellulose Derivatives

Cellulose is a homopolysaccharide with a linear long-chain and composed of β-D-glucopyranose units. It is the most abundant polymeric material from natural resources and is mainly found in plants [13]. As a material for food packaging, cellulose fibers have already been utilized to preserve fresh, dry, frozen, and liquid foods, as well as beverages—mainly in the form of cellophane, also known as regenerated cellulose films [14,19]. Cellulose fibers are mainly composed of cellulose, lignin, and hemicellulose, along with other extractives that are dependent on the source of the fibers [14]. They are also becoming an important and sustainable bio-based material in various industries due to their biocompatibility, chemical stability, non-toxicity, good barrier properties, and cheap price [14]. Cellulose fibers have outstanding mechanical properties, with high strength, high stiffness, and low density [24]. According to the tensile tests conducted by Bos H.L. et al., the tensile strength of a single elementary flax fiber—a relatively strong fiber compared to other fibers—is approximately 1500 MPa [25]. In addition, cellulose fibers are also very thermally stable. Investigations have shown that the presence of cellulose fibers has a positive impact on the thermal behavior of the films they create [24]. There are also a variety of cellulose derivatives, such as methylcellulose (MC) and hydroxypropyl methylcellulose (HPMC), which form strong and flexible water-soluble films [6]. Antimicrobial agents such as nisin can be embedded into the matrices of cellulose derivatives to inhibit the growth of bacteria. Zhuang et al. found that incorporating sorbic acid with HPMC film has a significant effect on inactivating *Salmonella montevideo* [26]. 

#### 2.1.4. Alginates, Carrageenan, and Agar

Alginates, carrageenan, and agar are all ionic polysaccharide-based gum biopolymers derived from seaweeds [6,27]. They have abundant free OH groups that make them polar and hydrophilic. Thus, these free OH groups can interact with water during the dissolution process to form the hydrogen bonds between the polymer chains, thereby creating films and coatings in the form of hydrocolloids. In addition, the ionic groups in alginates, carrageenan, and agar increase the compounds’ polarity, which affects their hydrophilic behavior [27]. 

Similar to other polysaccharide-based biopolymers, alginates, carrageenan, and agar are great materials for food packaging because of their biocompatibility, biodegradability, stable chemical properties, wide availability, low cost, good film-forming properties, and unique gum properties, including their gel-forming and thickening properties [28]. The edible films produced with alginates, carrageenan, and agar have already been applied for the preservation of a variety of meat and poultry products, as they exhibit outstanding gas and moisture barrier properties to prevent lipid oxidation and dehydration [28]. Previous research has shown that meat products preserved with gum polymer coatings and calcium alginate have improved sensory attributes—such as flavor and juiciness—compared to uncoated samples [29]. Alginates, carrageenan, and agar can be incorporated with other biopolymers to improve these properties. For instance, modified starches, oligosaccharides, or simple sugars can be combined with alginate films to enhance the tearing strength of the film [30]. Furthermore, it has been found that these gum-based films containing antimicrobial agents and chelating agents—such as lysozyme and ethylenediaminetetraacetic acid (EDTA)—are capable of inhibiting pathogens and spoilage bacteria [6]. 

### 2.2. Protein-Based Biopolymers

Protein-based biopolymers are derived from both animal and plant sources. Similar to carbohydrate-based biopolymers, their biodegradability, wide availability, and carrier ability allow them to incorporate other polymers and functional agents, such as antimicrobial agents. They are attracting increasing interest as biopolymers for food packaging [13]. Protein-based biopolymers also have good film-forming abilities and, due to their ordered and tightly packed hydrogen-bonded network structure, the protein-based films are great barriers against oxygen [28]. However, their hydrophilic nature and high moisture sensitivity result in poor water vapor barrier properties [31].

#### 2.2.1. Gelatin

Gelatin is a protein obtained from animal skin, connective tissues, and bone by partial hydrolysis of collagen, with a white-to-yellowish translucent appearance [30,32]. The properties of gelatin can be highly associated with factors such as the source (e.g., lactation gelatin, fish gelatin, or insect gelatin), the age of the animal, the type of collagen, and the processing methods used [33]. Gelatin has various applications in the pharmaceutical and food industries. It can be used to encapsulate ingredients and processed to be used as a coating for capsules and tablets [30]. In addition, gelatin has also demonstrated its potential as an edible film in food packaging to maintain food quality due to its water-binding, gel-forming, and film-forming abilities, strong gas barrier properties, non-toxicity, and swelling behavior in water [32]. However, gelatin films are still limited in food packaging applications due to their weak mechanical properties and water vapor barrier properties [11]. Therefore, plasticizers and modifications are essential for gelatin films to overcome their weak mechanical properties by increasing their toughness and decreasing their brittleness. In a study on the impacts of various plasticizers on gelatin films by Cao N. et al., plasticizers such as malic acid (MA) were found to improve the ductility of the films [34]. Polyethylene glycol (PEG) of different molecular weights could enhance mechanical properties such as tensile stress, while mannitol and sorbitol could improve the flexibility of the gelatin films. Gelatin modified with enzymes (e.g., transglutaminase) and other proteins (e.g., soy protein isolate) can improve thermal stability as well as water vapor resistance [35]. Furthermore, even water can act as a plasticizer for protein-based materials such as gelatin. Gelatin is also commonly integrated with other polymers to enhance films’ properties. For example, the polymerization of gelatin with polysaccharides such as chitosan can not only improve the film’s antioxidant and antimicrobial properties, it can also improve the film’s chemical and physical properties [36].

#### 2.2.2. Whey/Casein Protein

Milk protein concentrates (e.g., whey/casein proteins) can also be developed as materials for edible food packaging. Due to their functional properties and high nutritional value, milk proteins provide the additional benefit of forming an edible film material. Up to 80% of the protein in milk is casein [37]. On the other hand, whey proteins are mainly from the byproducts of the dairy industry—especially the cheese industry—helping to reduce the food industry’s waste [38,39]. Therefore, the wide availability of milk proteins contributes to the low cost of using milk them as packaging materials. Milk proteins have been found to have great film-forming abilities, depending on associative phase separation or coacervation mechanisms [37]. Whey protein isolates (WPIs) also have great film-forming abilities. Due to its random coil nature and the ability to form electrostatic, hydrophobic, and intermolecular hydrogen bonds with metal cations such as Na^2+^, Mg^2+^, and K^+^, casein can easily form films when combined with these metal cations in the form of caseinates. Much like other polysaccharide-based and protein-based materials, films formed by milk proteins exhibit low oxygen permeability and weak moisture resistance because of their hydrophilic behavior, but they have adequate mechanical, sensorial, and optical properties [38]. Due to the poor water vapor permeability of whey-protein-based antimicrobial films, research conducted by Ozdemir M. et al. investigated approaches to reducing their water vapor permeability [39]. As materials for antimicrobial food packaging, their poor water resistance caused dissolution in water and decreased the ability of the films to carry antimicrobial agents. The study showed that a mixture of 53% protein, 38% sorbitol, 8% beeswax, and 1% potassium sorbate would optimize the film’s characteristics, including minimizing the stickiness, water solubility, and water vapor permeability. In addition, the lower pH value of whey protein films containing p-aminobenzoic or sorbic acids can also contribute to the inhibition of microbial growth [40]. A study by Aisha and Abdullahi investigated the antimicrobial properties of whey protein isolate films that contained cinnamon essential oil [41]. While the film was ineffective against *Escherichia coli,* it was effective against *Staphylococcus aureus*. In a spoilage assay, the films successfully prevented fungal growth in bread, lowered lactic acid bacteria counts in chicken salami, and extended the shelf life of both foods. In addition, the whey protein–cinnamon oil films were biodegradable, degrading within 10 days under the action of soil microorganisms [41]. While there is little literature on the relationship between food rheology and whey protein isolate packaging, a film of sodium alginate, whey protein, and *Lactobacillus rhamnosus GG* did not impact the texture or the flavor of the bread [42]. More studies will need to be conducted on the relationship between the rheological properties of different foodstuffs and active packaging.

#### 2.2.3. Zein Protein

Zein is a protein derived from corn gluten with applications in edible food packaging and in forming nanofibrous layers [43]. Zein protein makes up almost 45–50% of the protein content in corn and is extracted by an aqueous–alcohol solution extraction process [44]. Although it is considered to be a byproduct of corn processing, limiting its applications, emerging research has demonstrated that its biodegradability and biocompatibility make it a suitable candidate for applications in food packaging. Moreover, several physical and chemical characteristics of zein, such as its excellent film-forming properties, make it superior to many other natural and synthetic alternatives—such as carnauba wax and shellac—as a coating agent [45]. Additionally, the water solubility of zein (except in alcohol solutions) creates an effective barrier against water vapor [44]. Furthermore, functional groups such as amines, amides, hydroxyls, carboxylates, and phenols allow various modifications to improve their characteristics and properties [45]. For example, zein films are naturally brittle, hard, and tough, but they can be modified with plasticizers [45]. Oleic and linoleic acids are natural plasticizers that are typically used when forming zein films [44]. Zein films can be formed by a wet process or a combined wet and dry process involving kneading, blowing, or extrusion equipment [45]. Despite being a byproduct of corn, zein is not readily produced because of the high cost of the aqueous–alcohol solution extraction process.

### 2.3. Lipid-Based Biopolymers

Lipid compounds are also becoming attractive as biopolymer materials for edible food packaging. The commercial use of lipid compounds as preservative coatings for fresh fruits and vegetables began in 1930 [30]. Compared to polysaccharide-based and protein-based biopolymers, the hydrophobic nature of lipid compounds contributes to exceptional water vapor barrier properties but weaker mechanical strength and oxygen barrier properties [28]. Therefore, these biopolymers are effective in retaining moisture in the food and can be incorporated with other polymers, such as polysaccharide-based materials, to enhance their mechanical properties. Some lipid-based biopolymers also play important roles as emulsifiers and surface-active agents [30]. Still, this review focuses on the polymers used as film-forming or coating materials.

#### Waxes

Waxes are classified as non-polar, highly hydrophobic lipids that can be dissolved in organic solvents, but not in bulk water [6]. There are currently a variety of applications of wax coatings in the preservation of fruits such as apples and citrus fruits (e.g., oranges, lemons, limes, mandarins, etc.) to reduce moisture loss and act as a microbial decontamination system. Recent studies have also explored the potential application of waxes combined with other antimicrobial agents to reduce microbial contamination and mold growth on poultry skins [46]. Studies show that the antimicrobial efficacy of wax can be affected by scald types. For instance, some waxes, such as carnauba wax, can effectively prevent the attachment of *Salmonella* to chicken skin, whereas beeswax does not have that ability. In addition, treatment with fumaric acid and calcium oxide (CaO) aqueous solution was found to enhance the antimicrobial properties of wax [47]. Water vapor and oxygen permeabilities were evaluated by Donhowe I.G. et al. for beeswax (BW), candelilla wax (CnW), carnauba wax (CrW), and microcrystalline wax (MW). [48]. The results showed that waxes possess excellent water vapor resistance with a low water vapor permeability value, but higher oxygen permeability compared to polysaccharide-based films such as cellophane. In addition, water vapor permeability and oxygen permeability are dependent on temperature. However, wax coatings on food can possibly result in anaerobic respiration and flavor changes because of elevated ethanol and acetaldehyde contents within the coatings [49].

## 3. Composites with Water and Oxygen Barrier Properties

In general, polysaccharide films offer good mechanical properties but poor moisture barrier properties [50]. A composite coating of starch and stearic acid on paper food packaging was found to have good moisture barrier properties with increased stearic acid concentrations. Another method to improve the moisture barrier properties is to incorporate nanoparticles into starch films [51]. The addition of 10% (*w*/*w*) cassava starch nanocrystals into starch films reduced water vapor permeability by 43% [52]. Gelled films formed from chitosan and a suitable crosslinker have decent water barrier and water resistance properties [53]. In addition, when chitosan is coated onto paper, it can reduce the moisture permeability without affecting the paper’s mechanical properties. Films of chitosan blended with cellulose or cellulose derivatives were found to enhance the shelf life of wheat bread and cheese, and they possessed several important properties such as transparency, high moisture resistance, and elasticity [53]. Cellulose-based nanomaterials are cost-effective, biodegradable, and have low environmental costs [54]. They are excellent oxygen barriers but poor moisture barriers, with water vapor permeability in the range of 2882 to 27,750 g·μm/m^2^ day kPa. The water vapor permeability of cellulose nanomaterial films increases with increasing humidity. Attempts to improve this property include acetylation, lignin, and esterification with hexanoyl and dodecanoyl chloride [54].

Films and coatings made from alginate are tasteless, odorless, and have low permeability to oxygen and other gases [55]. However, they suffer from poor moisture barrier properties due to the high hydrophilicity of alginate. The pure alginate films were reported to have a moisture content of 12.22% ± 1.37 and a water vapor permeability of 6.28 × 10 − 5 ± 0.24 g mm/m^2^ h Pa [56]. The addition of hydrolyzed cottonseed proteins into the alginate films was found to increase the water vapor permeability and film thickness, while maintaining the films’ biodegradability, solubility, oil barrier properties, and moisture content [56]. Likewise, the integration of CaCl_2_ into alginate matrices improved the water resistance and water vapor permeability, lipids could reduce water loss, and silver/montmorillonite nanoparticles improved antimicrobial activity and product shelf life [57]. Agar films also suffer from poor water barrier properties, poor thermal stability, and brittleness [57]. The incorporation of plasticizers and chitosan decreased the moisture barrier properties of the films, while lignin, soy protein isolate, nanocelluloses, nanosilver, and the nanoclay Cloisite Na+ improved the barrier properties [57].

In contrast, gelatin tends to swell or dissolve when in contact with food that has a high moisture content [58]. Different substances were studied for integration into gelatin films to improve their functional properties and their shelf-life-extending abilities. These properties can be improved through decreasing the intermolecular forces of protein chains, modifying the hydrophilicity of molecular structures, or forming strong covalent bonds with gelatin in films [58]. For example, the integration of green tea extract into gelatin decreased its water vapor permeability by 30% [59]. Lipids and oils also improved its water vapor permeability. Microcrystalline cellulose—a commercially available material prepared from wood fiber—was found to improve the water barrier and mechanical properties of soy protein–gelatin isolate films [60]. Milk-protein-based films also have the advantage of possessing several amino acid residues—mainly cysteine, which inhibits adventitious polyphenol oxidase in the packaged food—as well as the ability to constrain the enzymatic browning of products [61]. Whey-protein-based films have relatively better moisture barrier properties compared to other protein- and polysaccharide-based films but, due to their hydrophilic nature, their films lack good moisture barrier properties. The integration of zein into films improves their water vapor permeability. For example, the mixture of zein nanoparticles and whey protein reduced the water vapor permeability by up to 84% [62]. This is probably because of the hydrophobicity of zein nanoparticles and the interactions between the nanoparticles and polymer, which decreased the chain mobility and free volume of the film.

## 4. Antioxidant Packaging Systems

Oxidation can be introduced as a primary cause of food spoilage, and food quality may deteriorate while food components undergo oxidation. Lipid oxidation and protein denaturation are two significant causes of food deterioration. Antioxidants can effectively retard lipid oxidation and protein denaturation by minimizing the oxygen in food systems through the reduction capacity of active antioxidants [63]. An abundant number of researchers have put effort into limiting oxidation but, currently, the most applied solution is the direct addition of antioxidants to foods [64,65]. The drawbacks of this approach include the possibility of reduced antioxidant activity due to their interactions with food components during processing, along with the rapid decline in food quality after consuming the active compounds in the food [63,64].

**Figure 2:**
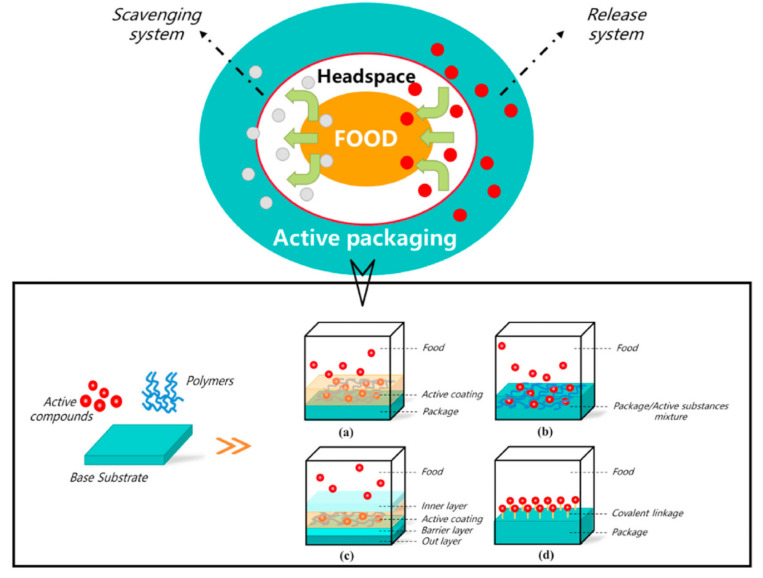
Main active antioxidant packaging systems (a) A releasing system in which active compounds are coated onto the surface of the packaging film (b) A releasing system in which active compounds are incorporated into the packaging film using polymers (c) A releasing system in which active compounds are incorporated into a different layer of the packaging films for more precise and controlled release (d) An oxygen scavenging system in which active compounds are covalently bound onto the surface of the packaging films(reprinted with permission from [63]).

The concept of active food packaging has been developed to overcome these two problems. Early forms of antioxidant packaging were in the format of sachets, pads, or labels. However, many chemicals in these systems are not edible, and if the pads or sachets carrying the chemicals are damaged, it may cause safety concerns. Thus, this problem might be prevented by applying safe and active packaging materials. This kind of packaging can be categorized into two forms based on antioxidant activity (Figure 2): The first form is the oxygen scavenging system, using materials such as iron or ferrous oxide. The second is the release system, in which antioxidants are released into food and eliminate free radicals [63,66]. Antioxidant compounds used in active packaging can be synthetic—such as butylated hydroxyanisole (BHA)—or natural, such as essential oils [63,65,67]. In recent years, many studies have been conducted on active packaging materials with antioxidants incorporated in the polymer matrix or on their surface [64,67,68].

Antioxidants can be physically or chemically incorporated into polymer films using methods such as surface coating, noncovalent incorporation, and covalent immobilization. In the physical incorporation, the antioxidant can be embedded into the polymeric matrix, adhered to the polymer film, or coated on the polymer matrix surface. Conversely, covalent bonds are recruited to bond the antioxidant to the polymer for chemical incorporation [63,64,66]. Moreover, the addition of inorganic NPs in biodegradable polymer-based matrices has been an efficient strategy to potentiate antioxidant effects in food applications [69].

Polymeric matrices are capable of retaining active compounds in their structure and releasing them appropriately. The polymers studied for active food packaging applications are either synthetic or natural products. Synthetic polymers have the advantages of low cost, favorable mechanical properties, heat resistance, and superior barrier properties. However, most synthetic films originate from non-renewable petroleum resources, are not biodegradable, and can potentially produce toxic compounds. Bio-based polymers could be introduced as a solution to the packaging waste problem. Natural bio-based polymers can also form edible and environmentally friendly films [63,70,71].

### Biopolymers as Carriers of Antioxidant Agents 

Recently, the use of biopolymers has been highly regarded, as this type of polymer is biodegradable, its recycling is straightforward, and it does not cause environmental damage and water pollution. The most important biopolymers used for active food packaging are biodegradable polymers obtained from synthetic sources such as PLA, PVA, and PCL, as well as natural ones such as proteins, polysaccharides, lipids, and their combinations [70,71]. Generally, biopolymer matrices based on polysaccharides and proteins have been used more than their peers in active food packaging because of their unique properties, such as being clean, renewable, non-toxic, edible, biodegradable, and biocompatible [69].

In 2021, Kadam et al. used pine needle extract as an antioxidant agent at concentrations of 5, 10, and 20%. The biopolymer used in this study was chitosan, while glycerin was utilized as a polymeric film plasticizer. The entrapment of the antioxidant in the polymeric matrix was carried out by adding it to the chitosan solution and subsequent film preparation. The results suggested that the films containing pine needle extract at 10 and 20% concentrations had high antioxidant properties and decreased oxygen permeability [72].

In 2019, Hu et al. designed a polymeric film based on Ginkgo Biloba extract (GBE) and gelatin. The GBE was added and entrapped in gelatin films developed by a casting technique. This group also investigated the impacts of the extract concentration on the film’s antimicrobial and physicochemical properties. It was claimed that the film’s antioxidant ability would be improved because of the active substances in the GBE [73]. Table 1 lists the major biopolymers used as matrices containing antioxidant agents.

## 5. Biopolymer-Based Composites with Antimicrobial Properties for Active Food Packaging

### 5.1. Metal and Metal Oxide Nanostructures with Antimicrobial Properties Integrated with Biopolymers for Active Food Packaging

**Figure 3:**
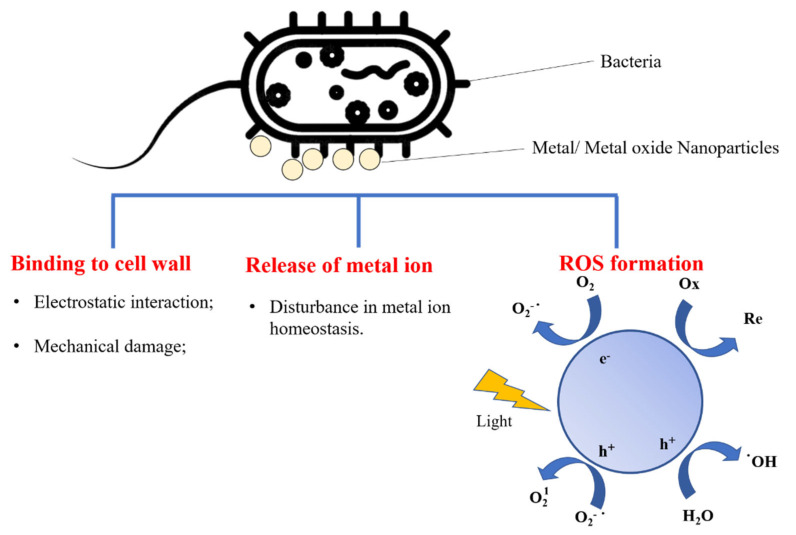
Molecular mechanisms of antibacterial activity of metal/metal oxide nanoparticles (ROS: reactive oxygen species).

Various metal- and metal-oxide-based nanomaterials—including CuO, Ag, ZnO, TiO_2_, and Fe_3_O_4_—have been investigated as antimicrobial agents. They have been integrated with biopolymers to form nanocomposite films for active food packaging. It is known that metal- and metal-oxide-based nanomaterials can cause cell death, mainly through alterations or destruction of the cell wall and enzymes, disruption of the nucleic acid pathways, disturbance of the metal ion homeostasis of bacteria, and the formation of reactive oxygen species (ROS) (Figure 3) [87,88,89]. In addition, the surface of bacteria is negatively charged at biological pH because of the existence of carboxylic acid groups in bacterial membrane proteins [88,90,91], which provide sites for binding positively charged metal oxide nanoparticles (MeO NPs) and metal cations. Lipopolysaccharides in the outer membranes of Gram-negative bacteria make their surface charge more negative than the surface charge of Gram-positive bacteria, leading to stronger electrostatic interactions between Gram-negative strains and MeO NPs [88,91,92].

TiO_2_ has been widely investigated in the active food packaging industry for its low cost, low toxicity to humans, and good antibacterial activity [93]. Electron–hole pairs can form on the surface of TiO_2_ NPs under ultraviolet light, and those electrons can trigger ROS formation to kill bacteria [94,95]. The antibacterial activity was obviously increased when TiO_2_ NPs were mixed or blended into film matrices, such as gelatin, chitosan, high-density polyethylene, and polylactic acid [96,97,98,99]. They were also a good barrier to UV by reducing light transmission, making them useful for food storage [98]. However, UV light is required to activate the photocatalyst of TiO_2_ NPs for ROS formation and killing the bacteria [100,101], limiting their application without light. ZnO is another candidate with commercial potential in active packaging because of its cheapness and high antimicrobial efficacy [102]. ZnO NPs can attack bacteria in various ways: (a) ROS formation under ordinary room light [103]; (b) induced penetration of the cell wall via electrostatic interactions between ZnO and bacteria, damaging the cell membrane and causing leakage [104]; or (c) inhibiting the action of respiratory enzymes, complex glycolysis, and transmembrane proton translocation through the existence of zinc ions [102,105]. ZnO NPs can be easily blended in the film matrix or coated on the surface during film processing. Chitosan, carboxymethyl cellulose, poly(vinyl chloride), polyurethane, and poly(lactic acid) films with ZnO NPs embedded in the matrix all showed enhanced antibacterial activity against either Gram-negative or Gram-positive bacterial strains [106,107,108,109]. Ag NPs have better antibacterial efficiency against a broad spectrum of microorganisms [110,111]. However, considering the cost of silver, Ag NPs are mostly used as additives to compensate for other materials or to enhance antimicrobial properties. For example, a matrix with Ag- doped TiO_2_ and ZnO NPs showed antibacterial ability with or without light [112,113]. The good UV barrier properties, non-toxicity, and high biocompatibility of Fe_3_O_4_ enhance its applications in food packaging [114]. Considering its insufficient antibacterial activity, Ag and sulfur were used to conjugate with Fe_3_O_4_ before forming films with carrageenan [115,116].

**Figure 4:**
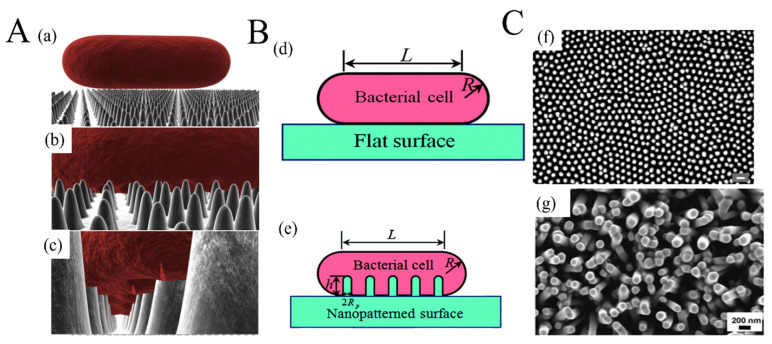
The rupture of bacterial cell walls by physicomechanical mechanisms: (**A**) Three-dimensional (3D) illustration of the interactions between a rod-shaped cell and the surface on which the nanorods grow vertically—(**a**) the contact between the cell and the surface; (**b**) the absorption of the cell onto the nanostructured surface; (**c**) the rupture of the cell membrane ((**a**–**c**) are reprinted with permission from [117]). (**B**) Schematic illustration of a bacterial cell adhering to (**d**) a flat surface and (**e**) a nanopatterned surface (reprinted with permission from [118]). (**C**) Comparison of the surface of clanger cicada wings and ZnO nanorods—(**f**) scanning electron micrograph (SEM) image of the surface of a cicada wing with (scale bar = 200 nm) (reprinted with permission from [117]); (**g**) SEM of the ZnO nanorods produced on polydimethylsiloxane by a hydrothermal method (reprinted with permission from [113]).

The applications of metal- or metal-oxide-based nanocomposites in food packaging are shown in Table 2. Most of the MeO NPs were directly mixed or blended with the polymer matrix, and their amounts in the composites varied from 0.5 wt% to 10 wt%. Although such a method is easy to operate, it was still considered to be less effective and economical than other alternatives. Because the antibacterial activity is mainly dependent on the contact surface between the food and the film, only the MeO NPs on the surface would directly interact with the bacteria. Therefore, using MeO NPs as the only coating on the substrate surface would be an effective way to achieve a balance between cost and effect. Interestingly, the bactericidal behavior could occur on naturally nanostructured surfaces, such as cicada wings [117]. The rupture of bacterial cell walls by physicomechanical mechanisms is shown in Figure 4A,B. There are many similarities between manmade nanostructured surfaces and cicada wings (Figure 4C). ZnO nanorods prepared by hydrothermal methods are grown oriented with around 100 nm in diameter [113]. They are similar to the nanopillar structure in cicada wings, either in orientation or dimensions. Morphology, size, and distribution of nanostructure are all important factors in designing antibacterial surfaces for food packaging.

**Table 2:** Metal and metal oxide nanostructures integrated with biopolymers for active food packaging.

Nanomaterials	Size/Distribution	Density	Matrix	Properties	Targeted Microorganisms	Ref.
ZnO	35.5–69.7 nm nanoparticles (NPs)	5 wt%	Chitosan and carboxymethyl cellulose films	Biodegradable coatings	*S. aureus*	[106]
	Around 200 nm NPs.	93.75 μg/cm^2^, 187.5 μg/cm^2^	Poly(vinyl chloride) film	UV irradiation required	*E. coli* or *S. aureus*	[107]
	<100 nm NPs.	0.05, 0.1, and 0.2 wt%	Hydroxyethyl cellulose and citric-acid-based biopolymer film	Good swelling abilities and hydrophilicity	*E. coli* or *S. aureus*	[108]
	30 nm NPs.	1–5 wt%	Polyurethane/chitosan composite film	Extended shelf life of food, improved mechanical properties, and reduced oxygen permeability	*E. coli* or *S. aureus*	[109]
	ZnO nanorods grow less directly (500 nm in diameter, 2–3 μm in length).		Poly(lactic acid)	Biodegradable	*E. coli* or *S. aureus*	[119]
ZnO + Ag	ZnO nanorods with vertical growth (160 nm in diameter, 2 μm in length).		Polydimethylsiloxane	No significant cytotoxicity	*E. coli* or *S. aureus*	[113]
ZnO + stearic acid	250–500 nm NPs.	2, 5 wt%	Isotactic polypropylene	Higher thermal stability and improved mechanical and impact properties	*E. coli*	[120]
TiO_2_	50–80 nm NPs.	10 wt%	Chitosan	Enhanced hydrophilicity and better mechanical properties; extended shelf life of fruit	*E. coli*, *S. aureus*, *Candida albicans*, *Aspergillus niger*	[96]
	0.2–0.3 μm	1 wt%	High-density polyethylene with CaCO3.	Extended shelf life of cheese	Inhibition of lactic acid bacteria and coliforms	[97]
	Nanotubes	0.5–5 wt%	Gelatin film	High UV barrier	*E. coli* and *L. monocytogenes*	[98]
	10 nm NPs	0.75 wt%	Polylactic acid nanofiber/film	Increased antibacterial activity under UV-A irradiation	*E. coli*, *S. aureus*	[99]
	12.22 nm	3.3–10 wt%	Alginate	Highly transparent to light	*E. coli*, *S. aureus*	[121]
TiO_2_+Ag	-	0.1–0.5%, *w/v*	Gelatin–chitosan film	Reduced light transmittance; antibacterial ability with or without light	*E. coli*, *S. aureus*, *Botrytis cinerea*	[112]
Au-TiO_2_	Au NPs with TiO_2_ as shell 45 nm	2.5 wt%	Alginate nanocomposite	Excellent visible light absorption; increased production of reactive oxygen species	*E. coli*, *S. aureus*	[122]
Ag	13.7 ± 3.5 nm	0.075–0.3 wt%	Carrageenan	Improved UV shielding properties	*E. coli* and *L. monocytogenes*	[123]
Fe_3_O_4_+Sulfur	281.4 nm NPs	0.5–1.0 wt%	Carrageenan	Effectively blocked UV light; improved thermal stability	*E. coli* and *L. monocytogenes*	[115]
Fe_3_O_4_-Ag	-	2 wt%				[116]
Fe_2_O_3_/TiO_2_	10–27 nm	5–20 wt%	Chitosan, polyvinyl alcohol	Enhanced antibacterial activity and mechanical properties	*E. coli*, *S. aureus*	[124]
CuO	-	2 wt%	Carrageenan	UV barrier and strong antibacterial activity	*E. coli* and *L. monocytogenes*	[125]

### 5.2. Natural Products with Antimicrobial Activity Integrated with Biopolymers for Active Food Packaging

Biopolymers are biodegradable and sourced from renewable materials, giving them great potential for food packaging applications [126]. Starch and cellulose are both abundant in nature [126,127,128]. Their abundance, low cost, and edibility make it feasible to use them in the food industry. Cellulose acetate can be easily dissolved in acetone up to 10% (*w*/*v*), and the film is formed after solvent evaporation [129]. These films show good elongation and puncture resistance, but their intrinsic biocompatibility and non-toxicity make their antimicrobial activity less sufficient. The investigation of biopolymers with enhanced properties has attracted attention in recent years.

The addition of antimicrobial agents (e.g., MeO NPs; naturally derived and synthetic molecules or polymers) to the biopolymer matrix is an effective approach to fight against bacteria. As mentioned in the previous section, the antibacterial activity can be greatly improved with the addition of TiO_2_, Ag, and reduced graphene oxide nanostructures into matrices, such as starch-based films [97,130,131]. Ag can also enhance the films’ resistance to oil, while TiO_2_ is able to reduce light transmission. Similarly, the addition of these MeO NPs into matrices of carrageenan—a sulfated linear polysaccharide extracted from red seaweeds of the Rhodophyceae class [132]—could enhance their antimicrobial efficiency [115,123,125,133]. Notably, CuO NPs and Ag NPs both increased their mechanical strength, while CuO NPs even increased their barrier properties against water vapor, which are related to inter- and intramolecular interactions between the polymer chains and NPs [125].

The incorporation of essential oils with biopolymers has also been reported to exhibit antibacterial properties. Essential oils are lipophilic compounds extracted from plants, the components of which contain phenols, aldehydes, ketones, terpenes, etc. [134]. The lipophilicity and phenolic components of essential oils allow them to easily penetrate bacterial cell membranes, causing the leakage of ions and contents of cells, followed by the disruption of lipid–protein interactions [135,136]. Starch-based films containing various essential oils—including cinnamon, lavender, peppermint, cumin, and so on—all exhibited enhanced antibacterial activities against both Gram-positive and Gram-negative bacteria, along with fungistatic effects on mold, extending the shelf lives of fruits and meatballs [137,138,139,140,141,142]. Carrageenan and carboxymethyl carrageenan both showed bacteriostatic effects against *S. aureus*, *B. cereus*, *P. aeruginosa*, and *E. coli*, because of the acidic pH environment caused by the sulfate residues and carboxylate groups [143,144]. However, the anionic charges of these carrageenans may have restricted interactions between the polymers and negatively charged bacterial cell membranes, reducing their bacteria-killing efficiency. Compared to starch-based films, encapsulation of carrageenan with essential oils and other plant extracts (such as olive leaf extract) showed enhanced bacteriostatic effects [145,146,147].

Other bioactive reagents, such as polyhexamethylene biguanide (PHMB) and epsilon-polylysine (as is shown in Table 3), are cationic polymers with broad antibacterial activities. These properties have proven to be affected by the incorporation of such polymers into carrageenan, starch, and starch/ cellulose film matrices [148,149,150]. The complex film of starch and cellulose can totally inhibit *E. coli* when the PHMB amounts to over 6%, prolonging the shelf life of fruit [149]. In addition, lytic bacteriophages can be loaded in the cellulose matrix for enhanced antibacterial activity, and they can be viable on the matrix for 14 days [129]. Rosin-modified cellulose can work as a reinforcement filler in other matrices, such as polylactic acid and chitosan, for optimized mechanical properties [151]. Rosin in cellulose and chitosan can contribute to synergistic antimicrobial effects against *E. coli* and *B. subtilis.*

On the other hand, the encapsulation of bioactive reagents with antibacterial properties into the biopolymer matrix may lead to the liberation of reagents from the matrix and their migration to food. The potential toxicity of these reagents may pose food safety concerns. The effectiveness of these composites on bacteria may also decrease as the release of effective ingredients increases. Therefore, it might be necessary to find another solution to design and modify existing natural polymers and their derivatives to obtain intrinsic antibacterial properties, such that there is persistent effectiveness against bacteria in the long term, reducing the release of potential toxins into the food.

Chitosan and its derivatives are applied as raw materials for food packaging because of their biodegradability, biocompatibility, and antimicrobial ability against various strains [21,152]. The mechanism of antimicrobial activity comes from the protonated and methylated amine groups on the chitosan, which cause the whole polymer to exhibit positive potential and have relatively strong electrostatic attractions with anionic groups and the negatively charged cell membranes of microbes [153]. However, the antibacterial activity and water solubility of chitosan would be reduced in neutral aqueous media. Furthermore, the amino groups of chitosan can be modified to form quaternary ammonium groups with methylating reagents [154]. The quaternary ammonium salt can also be obtained through the formation of 2-N-Hydroxypropyl-3-trimethylammonium chloride chitosan (HTCC), which was reported to have enhanced antibacterial effects against *E. coli*, *S. aureus*, and *Botrytis cinerea*, with inhibition rates of up to 99% [155]. These quaternary ammonium salts tend to have a more positively charged surface and stronger interactions with bacterial membranes than unmodified chitosan, promoting the leakage of cell contents and killing the bacteria as shown in Table 3 [156].

In addition, it has been reported that divalent cations such as Mg^2+^ and Ca^2+^ are essential for the stabilization of polyanionic lipopolysaccharides on the outer membranes of Gram-negative bacteria [157]. The chelation of these metal ions onto the cell membrane can influence the permeability of the membrane and disrupt the transportation of nutrients, representing another strategy to increase the antibacterial activity. Therefore, this method has proven useful in forming Schiff bases—a type of good chelating reagent for metal ions—on the biopolymer chains to obtain inherent antibacterial properties. The amino groups in the chitosan chains act as reactive sites to form Schiff bases (–RC=N–) by reacting with aldehydes and ketones, such as cinnamaldehyde, salicylaldehyde, ethyl vanillin, and citral [158,159,160,161]. These modified biopolymers all exhibit inhibitory effects on Gram-negative and Gram-positive bacteria. The antibacterial activity decreases with the increasing strength of electron-withdrawing groups on the aromatic ring [162]. Interestingly, Schiff-base-based films made from chitosan and ethyl vanillin showed higher inhibitory efficiency against *E. coli* than against *S. aureus*, which is consistent with the antibacterial mechanism [160].

**Table 3:** The application of biopolymers with antibacterial activity for food packaging.

Biopolymers	Strategies	Effect	Ref.
Cellulose	Incorporation with rosin	Antibacterial activity against *E. coli* and *B. subtilis*	[151]
	Mixing with lytic bacteriophages	Bacteriophages remained viable for 14 days	[129]
Starch	Addition of essential oil (lemon)	Enhanced antibacterial activity against *S. aureus* and *E. coli*	[137]
	Cinnamon essential oil	Against *Salmonella Typhi*, *S. aureus*, and *E. coli* (suitable for meatball packaging); extended shelf life from 48 h to 96 h for pork	[138]
	Lavender essential oil	Against *S. aureus* and *E. coli* (not suitable for food with a high-water content)	[139]
	Peppermint and lime oil	Inhibited growth of mold; delayed ripening during mangosteen fruit transportation	[140]
	Cumin essential oil	Reduced rot lesion on infected pears caused by *Alternaria alternata*	[141]
	Carvacrol and thymol essential oils	Fungistatic effect against *Colletotrichum gloeosporioides* on mango and papaya; reduced the incidence of anthracnose symptoms; extended shelf life from 4–5 days to 8 and 13 days	[142]
Carrageenan	Encapsulation of orange essential oil and trehalose	Showed resistance to Gram-positive bacteria	[145]
	Encapsulation of olive leaf extract	Reduced the count of psychrophiles five times further than commercial films for lamb meat packaging	[146]
	Fabrication with epsilon-polylysine	Broad antibacterial activity; inhibited growth of *A. acidoterrestris* in juice	[148]
Starch and cellulose	Polyhexamethylene biguanide (PHMB)	Prolonged shelf life of grapes; inhibition rate against *E. coli.* reached 100% when PHMB% > 6%	[149]
Starch		Better efficacy against *B. subtilis* than against *E. coli*	[150]
Chitosan	Formation of quaternary ammonium salt (N,N,N-trimethyl-chitosan chloride)	Higher inhibition efficiency on *E. coli*	[154]
	Formation of quaternary ammonium salt (N-(2-hydroxypropyl)-3-trimethylammonium chitosan chlorides) (HTCC)	Inhibition rate of *E. coli*, *S. aureus*, and *Botrytis cinerea* up to 99%; prolonged shelf life of strawberries by over 5 days	[155]
		Larger inhibition zone as HTCC content increases; extended shelf life of bananas by over 5 days for	[163]
	Functionalization with cinnamaldehyde	Sustained release of active reagents; enhanced fungicidal effect on *R. stolonifer* in bread slices	[158]
	Modification by salicylaldehyde/TiO_2_	Full eradication of *S. aureus* and *P. aeruginosa*	[159]
	Formation of Schiff bases containing halogenobenzenes	>95% inhibition of *Botrytis cinerea*	[164]
	Formation of Schiff bases containing benzaldehydes	Electron-withdrawing group on aromatic ring decreases the antibacterial activities	[162]
	Formation of Schiff bases containing ethyl vanillin	An excellent barrier to UV light; higher inhibitory efficiency against Gram-negative bacteria.	[160]

## 6. Active Food Packaging Made of Composites to Control Carbon Dioxide

In general, CO_2_ is beneficial for food protection, as a high amount of CO_2_ can be beneficial for preventing microbial growth in food products and their oxidation and maintains the freshness of food by suppressing physiologically reactive deterioration such as respiration, ethylene production, etc. However, the level of CO_2_ in the packaging’s atmosphere should be controlled, as the benefit of CO_2_ is totally different for various products. For some products, such as dairy products, high concentrations of CO_2_ can contribute to shelf-life extension; however, too much CO_2_ accumulation in the packaging may also cause undesirable changes in product quality, such as discoloration and adverse effects on the flavor and texture [165,166].

The most important factor that should be considered for the design of packaging is the CO_2_ gas permeability of the packaging layer. For instance, for packaging with high permeability to CO_2_, using a CO_2_-emitting system might be essential to reduce the respiration rate and microbial growth. CO_2_ produced from foods is one of the crucial parameters for designing active food packaging to control CO_2_ levels, as the release of CO_2_ produced from fresh food may cause the packaging to burst. This released CO_2_ can be controlled using a CO_2_ scavenger. As a result, it is reasonable to assume that the rate and amount of CO_2_ to be absorbed or released should be balanced based on certain criteria, such as the permeability of the packaging layer, the production kinetic of CO_2_ by food, and its dissolution in food [167,168,169]. CO_2_-emitting systems are used in the food packaging industry to increase CO_2_ concentrations, leading to the suppression of microbial proliferation on the surface of food and preventing shrinkage or collapse of semi-rigid plastic packages. On the other hand, recruiting CO_2_ scavengers is undeniably important in some food packaging systems. Active materials, including metal oxides, metal hydroxides, and physical gas adsorbents accompanied by polymers, can be used as CO_2_ absorbent systems [167,170]. Table 4 lists the different CO_2_ absorbers and emitters, including the commercial ones used for active food packaging.

### 6.1. CO_2_ Emitters

The idea of using a CO_2_-emitting system was adapted to active food packaging to maintain high levels of CO_2_ during storage to further inhibit microbial growth, provide smaller package volumes, prolong shelf life, and prevent the deformation of packaging, as this could play a role in compensating for the absorption of CO_2_ into the food product in the early storage stages [179].

The emitters are generated as a pad, sachet, or combined liquid absorber. These systems are generally composed of ferrous carbonate, ascorbic acid, sodium bicarbonate, or a mixture of sodium bicarbonate and citric acid. Some examples of commercial carbon dioxide emitters include CO_2_^®^ Fresh Pads and UltraZap^®^ XtendaPak pads, the latter of which are a combination of a CO_2_ emitter and an antimicrobial pad. NaHCO_3_ and citric acid are common ingredients in CO_2_-emitting systems. When the liquid makes contact with the NaHCO_3_–citric acid system, the pH drops, and CO_2_ is emitted into the packaging. Another type of emitter used in active packaging is ferrous carbonate (FeCO_3_), which releases CO_2_ upon its dissolution in acidic environments [180]. Hansen et al. tested a CO_2_-emitting pad that was prepared by adding NaHCO_3_ and citric acid to an absorber pad. The pad was tested along with a modified atmosphere packaging to determine the effects of CO_2_ emitters on shelf life in a vacuum and in low-headspace MAP. It was found that the emitter improved shelf life in both a vacuum and MAP but did not alter the bacterial composition in the packaging [171].

### 6.2. CO_2_ Absorbers

CO_2_ scavengers are commercially available in sachet, pouch, and label forms in food packaging systems. Generally, CO_2_ absorbers perform three substantial purposes: (1) reducing the excess pressure and volume expansion of packages of CO_2_-producing food, (2) precise control of CO_2_ levels in fresh produce packaging, and (3) protection of iron-based oxygen scavengers [167].

CO_2_ absorption can be achieved through different processes, such as chemical reactions, membrane separation, cryogenic condensation, and physical adsorption. However, chemical reactions and physical absorbers have been considered to be the most important methods in CO_2_-scavenging systems [166,167].

#### 6.2.1. Physical Adsorption of CO_2_

The physical adsorption of CO_2_ gas can be processed using materials such as zeolite and activated carbon. Physical adsorption is reversible in nature, and the equilibrium is directly affected by environmental conditions. For physical adsorption, the absorbers’ microporous structure—including the properties of pore volume, size distribution, and surface area—plays a major role in CO_2_ adsorption. Generally, the absorbers are in the form of sheets, blocks, powder, beads, granules, or packed in a porous pocket or sachet. As the normal adopted pressure in food packages is less than 1 atm, the capacity for physical absorption is quite low in comparison with chemical absorbers [167,181].

#### 6.2.2. Chemical Absorption of CO_2_

Although many alkaline solutions can potentially be used for CO_2_ removal, calcium hydroxide is the most common CO_2_ scavenger in food packaging systems. The reaction between Ca(OH)_2_ and CO_2_ is highly spontaneous and produces calcium carbonate and water as byproducts [182]. In addition to calcium hydroxide, some other chemicals—such as sodium carbonate, sodium glycinate, and calcium oxide—have been utilized for CO_2_ absorption. Among them, sodium carbonate can be utilized for CO_2_ scavenging in special conditions, such as foods with high moisture content. Calcium oxide is usually used for CO_2_ absorption when large volumes of fresh products are transported by vehicles, but it is not utilized in smaller packages [181,182].

It is also possible to use biopolymers or their incorporation with another material for CO_2_ removal. For instance, it is claimed that the D-glucosamine element of chitin has intrinsic properties for CO_2_ absorption, and this biopolymer has been introduced as a good candidate for CO_2_ removal [181]. Furthermore, Wang et al. prepared a type of biofilm based on agar incorporated with sodium carbonate (SC) and/or sodium glycinate (SG) as a CO_2_ absorber and used it for 200 g of mushrooms in a porous bag at 10 °C. The advantage of these absorbers was confirmed by observing low bacterial counts, firmer texture, less color change, good taste, and reduced humidity in the packaging. Moreover, the results suggested that using the SC film contributed to the highest CO_2_ absorption, followed by the SC/SG and SG films [183].

## 7. Conclusions

Active food packaging made of biopolymer-based composites is an innovative alternative to conventional plastic packaging to reduce recycling issues and prevent exclusive utilization of unrenewable petroleum resources. Antimicrobial packaging systems have become increasingly attractive to reduce the risk of foodborne illnesses, beyond acting as a physical barrier to prevent food contamination. Polysaccharide- and protein-based biopolymer films possess better mechanical and gas barrier properties compared to lipid-based biopolymer films, whereas lipid-based biopolymer films have better water vapor barrier properties. Various treatments or combinations with other polymers and plasticizers could effectively improve these properties as well as overcome the exhibited weaknesses. Metals and metal oxides, bioactive biopolymers, and polyphenols are all common antimicrobial agents used for active food packaging. Meanwhile, the current progress in the development of active food packaging for maintaining food quality by controlling CO_2_ levels has been reviewed. Furthermore, new strategies for developing biopolymer-based composites with strong mechanical properties should be investigated in future works. Although additives in the composites show effective antimicrobial activities, the optimization of the composition is important to achieve the balance between cost and effects. The inhibition of the migration of additives into food should also be studied for food safety concerns.

## Figures and Tables

**Table 1:** Biopolymer-based matrices containing antioxidant agents for active food packaging.

Biopolymer-Based Matrix	Antioxidant	Effect	Ref.
Gelatin/starch	Corn stigma extract (natural)	Increase in bioactive and antioxidant properties; reduction in lipid oxidation by 60% after the incorporation of the corn stigma extract in the polymer matrix	[74]
Chitosan–gum arabic edible film	Cinnamon oil (natural)	Significant enhancement of antioxidant effectiveness; enhancing water barrier properties	[75]
Chitosan	Tea polyphenols (natural)	Microbiological shelf-life extension; reduction in lipid oxidation and discoloration; maintaining acceptable sensory quality	[76]
Polylactic acid (PLA)	Synthetic phenolic antioxidants (SPA) (synthetic)	The high release rate of the antioxidant agent; reduction in the amounts of directly added antioxidants in foods	[77]
Polyvinyl alcohol (PVA)/corn starch	Pineapple peel extract	Improved antioxidant activity of the developed film as compared to the control film	[78]
Zein fibers	Yerba mate extract (natural)	Much higher antioxidant activity for zein fibers loaded with a 5 wt% extract	[79]
Furcellaran and gelatin (FUR/GEL)	Pu-erh (RTE) and green tea (GTE) extracts	Improved antioxidant activity and antimicrobial properties of FUR/GEL with GTE	[80]
Guar gum/carboxymethyl cellulose incorporated with halloysite nanotubes (HNTs)	Litchi shell extract (LSE)	Increased antioxidant activity; increased UV light barrier properties	[81]
Licorice residue extract (LRE)	Soy protein isolate (SPI)	Great antioxidant activity; excellent UV barrier properties	[82]
Sodium alginate	Essential oils (EO) of *R. officinalis* L., *A. herba-alba* Asso, *O. basilicum* L., and *M. pulegium* L.	Improved antioxidant activity; decreased moisture thickness and tensile strength; strong antibacterial properties	[83]
Polyethylene (PE) films coated with chitosan and the liposome loaded with LEO and silver nanoparticles (PC-Lip/LEO/Ag NPs)	Laurel essential oil (LEO)	Good antioxidant properties and antimicrobial activity; strong antimicrobial activity; extended storage period from 9 days to 15 days at 4 °C	[84]
Olive leaf extract	Carrageenan	High antioxidant activity; reduction in tensile strength; high water vapor permeability; good barrier properties	[85]
Noni (*Morinda citrifolia*) fruit polysaccharide (NPS)	Blueberry leaf extract (BLE)	Increased antioxidant activity of the films; greater water vapor permeability	[86]

**Table 4:** Applications of CO_2_ emitters and absorbers in active food packaging.

System Type	Food	Strategy	Effect	Ref.
CO_2_ emitter	Cod loins (farmed Atlantic cod, *Gadus morhua*)	NaHCO_3_ and citric acid	Improvement of initial freshness; shelf-life extension with reduced microbial growth	[171]
CO_2_ emitter	Chicken	NaHCO_3_ and citric acid	Reduction in drip loss; the inhibition of microbial growth and avoidance of packaging collapse; extension of sensory and microbial shelf life	[172]
CO_2_ emitter	Gutted sea bass	McAirlaid’s Inc.^®^ (commercial)	Shelf-life extension with reduced microbial growth	[173]
CO_2_ absorber	Pear	Ageless^®^ (commercial)	CO_2_ levels reduced in the bags during cold storage and preventing the development of internal browning	[174]
CO_2_ absorber	Eggplant	Lipmen^®^ (commercial)	Inhibition of fruit deterioration in a broad storage temperature range; reduction in chilling injury	[175]
CO_2_ absorber	Mushroom	Ca(OH)_2_ (chemical absorption)	Improving mushroom preservation; reducing yeast/mold growth and decay	[176]
CO_2_ absorber	Kimchi	Zeolite (physical adsorption)	Inhibition of volume expansion and pressure buildup	[177]
CO_2_ absorber	Kimchi	Ca(OH)_2_/zeolite (combination of physical adsorption and chemical absorption)	Solving volume expansion problems and breakage of the package without affecting the kimchi’s ripening	[178]

## Data Availability

Not applicable.
